# Experimental Implementation of Automatic Control of Posture-Dependent Stimulation in an Implanted Standing Neuroprosthesis

**DOI:** 10.1155/2019/2639271

**Published:** 2019-03-14

**Authors:** Brooke M. Odle, Lisa M. Lombardo, Musa L. Audu, Ronald J. Triolo

**Affiliations:** ^1^Department of Biomedical Engineering, Case Western Reserve University, Cleveland 44106, USA; ^2^Motion Study Laboratory, Louis Stokes Cleveland Veterans Affairs Medical Center, Cleveland 44106, USA

## Abstract

Knowledge of the upper extremity (UE) effort exerted under real-world conditions is important for understanding how persons with motor or sensory disorders perform the postural shifts necessary to complete many activities of daily living while standing. To this end, a feedback controller, named the “Posture Follower Controller”, was developed to aid in task-dependent posture shifting by individuals with spinal cord injury standing with functional neuromuscular stimulation. In this experimental feasibility study, the controller modulated activation to the paralyzed lower extremity muscles as a function of the position of overall center of pressure (CoP), which was prescribed to move in a straight line in forward and diagonal directions. Posture-dependent control of stimulation enabled leaning movements that translated the CoP up to 48 mm away from the nominal position during quiet standing. The mean 95% prediction ellipse area, a measure of the CoP dispersion in the forward, forward-right, and forward-left directions, was 951.0 ± 341.1 mm^2^, 1095.9 ± 251.2 mm^2^, and 1364.5 ± 688.2 mm^2^, respectively. The average width of the prediction ellipses across the three directions was 15.1 mm, indicating that the CoP deviated from the prescribed path as task-dependent postures were assumed. The average maximal UE effort required to adjust posture across all leaning directions was 24.1% body weight, which is only slightly more than twice of what is required to maintain balance in an erect standing posture. These preliminary findings suggest that stimulation can be modulated to effectively assume user-specified, task-dependent leaning postures characterized by the CoP shifts that deviate away from the nominal position and which require moderate UE effort to execute.

## 1. Introduction

Spinal cord injury (SCI) often results in partial or total paralysis of the trunk and lower extremity (LE) muscles. Implanted neuroprostheses (NPs) utilizing functional neuromuscular stimulation (FNS) can restore basic standing function in individuals with SCI, providing them with the independence to accomplish several activities of daily living [1, 2]. Standing NPs supply constant preprogrammed open-loop stimulation to the trunk, hip, and knee extensors to maintain a single, upright stance. Thus, to maintain balance in the presence of postural perturbations, NP users rely on voluntary upper extremity (UE) effort exerted on a support device, such as a walker or a countertop. To address this limitation, previous groups explored closed-loop feedback control systems for standing with stimulation employed at individual joints [3–8] as well as a stimulation controller based on comprehensive or global joint feedback combined with center of mass (CoM) acceleration that rejected destabilizing perturbations and reduced the UE effort to maintain standing balance [9]. However, these advanced control systems have been designed to maintain only a single upright setpoint in the nominal standing position. Users are only able to stand optimally and resist potentially destabilizing perturbations in one erect, neutral posture rather than at forward- or side-leaning postures best suited for specific functional tasks [10, 11]. To adjust posture away from the erect stance with existing systems that deliver preprogrammed patterns of stimulation, users must exert voluntary UE effort to push or pull against the support device. At these new positions, the patterns of stimulation tuned for erect standing become suboptimal and may over or understimulate the muscles required to maintain the new task-dependent postures.

One benefit to the ability to adjust posture from the erect stance is the ability to prepare for a functional task, such as reaching and manipulating objects on shelves. This also gives users the ability to reach the full extent of their standing workspace, thereby providing them with greater independence and access to objects in the environment. Another benefit is adjusting posture laterally, to rest muscles on one side of the body as a means of mitigating fatigue and prolonging overall standing times. These benefits are further supported by the work of Abbas and Gillette [12], which suggested that shifting posture in the anterior-posterior (AP) and medial-lateral (ML) directions would be critical in enabling standing NP users to accomplish various functional tasks.

To achieve these changes in posture, the location of the projection of the total body CoM on the base of support will have to change smoothly and continuously. Thus, control systems that can automatically maintain standing balance should include CoM position feedback and modulate stimulation to the LE muscles as posture is adjusted away from the erect stance. One such system (the Posture Follower Conftroller or PFC) was developed and tested in simulation [10]. In that simulation feasibility study, the model exerted voluntary UE effort to manually adjust the CoM location from an initial neutral setpoint towards the new desired forward- or side-leaning posture, while the controller continually updated the neural stimulation to maintain activations that were optimal for each change in position. Controller performance was measured with respect to (1) the UE effort, defined as the UE forces exerted as the simulated user changed postures and (2) the ability to track a moving object with the CoM as the object moved in the forward, diagonal, and lateral directions. The PFC reduced UE effort by an average of 50%, compared to using the UEs alone. In general, CoM tracking with the PFC and UE alone was similar, except for one instance of an overshoot when moving in the left-diagonal direction without the active controller. These encouraging simulation findings were encouraging and support the development of posture-dependent control of stimulation for a standing NP.

The next step in the development and deployment of posture-dependent control systems is to implement the PFC in the laboratory environment with a standing NP user. The simulation results in [10] used an ideal UE controller that modulated UE forces as a function of shoulder displacement and velocity away from the erect posture position. The actual UE forces exerted by a standing NP user are generally obtained from measuring devices in the laboratory and could vary considerably between individuals. Knowledge of the actual UE effort required by standing NP users to change posture will greatly help in the design of control systems for ensuring balance during performance of important activities of daily living.

The aims of this work are (1) to explore the effectiveness of a PFC to enable NP users to lean away from the erect posture and (2) to examine the contribution of UE effort to leaning postures in real subjects with SCI using a NP for standing. In this exploratory study, standing performance was determined by the following metrics: (1) maximum resultant UE effort contributed to leaning movements in the AP and ML directions as LE muscle activation is modulated and (2) CoP-tracking deviations from a prescribed straight-line path.

## 2. Materials and Methods

### 2.1. Subject and Standing Neuroprosthesis System

A 27-year-old male with motor incomplete C5 tetraplegia (AIS C) participated in the experiments. He was approximately 185.4 cm tall and weighed 58.5 kilograms when the experiments were conducted. He received a 16-channel implanted LE NP one year prior to data collection and was a regular user for reconditioning exercise and standing. At the time of the study, he could stand quietly in the neutral position for 25 minutes with 93% body weight (BW) supported by his legs, utilizing UE effort only for a light touch on the walker to maintain balance. Prior to participating in the experiments, the subject signed informed consent forms approved by the Institutional Review Board of the Louis Stokes Cleveland Veterans Affairs Medical Center.

The standing NP consisted of one surgically implanted 16-channel stimulator telemeter [13]. The implanted system targeted the following muscle groups: hip extensors (Gluteus Maximus (GMX), Hamstring (HM), and posterior portion of the Adductor Magnus (PA)), hip abductors (left Gluteus Medius (GMED)), trunk extensors (Lumbar Erector Spinae (ES)), and the trunk lateral benders (Quadratus Lumborum (QL)). A selective, multicontact, flat interface nerve cuff electrode [14] was implanted on the proximal femoral nerve near the inguinal ligament, to activate the three uniarticular vasti muscles of the quadriceps (QD) while avoiding recruitment of the sartorius and the biarticulated rectus femoris, which induce hip flexion that compromises erect neutral standing. All other muscles were activated with surgically implanted intramuscular electrodes [15]. Pulse amplitudes (0.8, 2.1, 18, or 20 mA) were set on a channel-by-channel basis while pulse duration (0-250 *μ*s) and frequency (0-20 Hz) were modulated independently on a pulse-by-pulse basis on each channel to achieve the desired motion. A rechargeable wearable external control unit (ECU) [16] delivered power and command signals to the implanted pulse generator via a close-coupled inductive link maintained by a transmitting coil taped to the skin over the stimulator. The ECU coordinated the delivery of temporal patterns of stimulation through all 16 channels simultaneously.

To target the right GMED for postural control in the ML direction and bilateral tibialis anterior (TA) and gastrocnemius (GS) for postural control in the AP direction, the implanted stimulation system was supplemented with self-adhesive surface electrodes. These muscles were recruited because they were not available in the subject's implanted system. Surface stimulation was delivered at a constant frequency of 20 Hz, variable pulse width up to 250 *μ*s dependent on controller output, and a fixed pulse amplitude (100 mA for the right GMED and bilateral GS, and 30 mA for the bilateral TA).

Real-time control of stimulation was implemented with a custom software developed in MATLAB/Simulink R7.9 and the xPC Target toolbox (MathWorks Inc., Natick, MA). A Windows (Microsoft Inc., Redmond, WA) host computer was utilized to build customized applications, while a dedicated (target) computer with the Pentium Dual-Core 3 GHz microprocessor (Intel Inc., Santa Clara, CA) with 2 GB of RAM was responsible for running the applications in real time. The host and target computers communicated via the TCP/IP protocol. Data were acquired using a NI PCI-6071E board (National Instruments Inc., Austin, TX). For the experiments described, all real-time controller and stimulation parameters were sampled at 40 Hz. The stimulus values for erect standing were determined by clinical observation whereby the subject exhibited ample knee, hip, and trunk extension to achieve an erect posture without discomfort. Baseline standing stimulation values are listed in Table 1.

### 2.2. Posture Follower Controller Design

**Figure 1:**
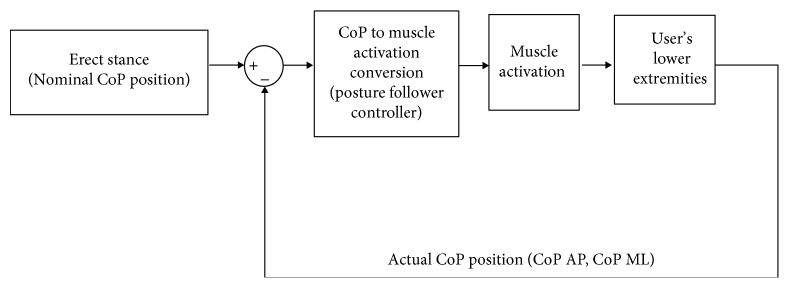
Control setup. The user stands erect on force plates, which measure the center of pressure (CoP) position in the anterior-posterior (AP) and medial-lateral (ML) directions. The user leans away from the erect stance, adjusting the overall CoP position towards the ends of the paths in the forward and diagonal directions. The force plates continuously track the resulting changes in the CoP position and the posture follower controller converts the changes in the CoP position to muscle activation, which is applied to the lower extremities.

In this study, the overall center of pressure (CoP) position (a function of the location of the vertical ground reaction force vector) was used as the feedback signal for the PFC. A more suitable feedback signal would be the orthogonal projection of the whole-body CoM or center of gravity (CoG). However, there are challenges in implementing the CoM position (a function of the location of the total body mass, as the feedback signal). Currently, there is no means for the quantity to be computed or estimated from body-mounted sensors in real time rapidly and accurately enough to use as a stimulus control signal with a paralyzed user. The CoM, CoG, and CoP are equivalent during static conditions. Thus, the overall CoP position was used as a surrogate because it can be readily obtained from two force plates (AMTI, Watertown, MA) in the laboratory, making it a more practical control signal for this exploratory study. The laboratory-based PFC took the form of a proportional feedback controller, so it tracks voluntary changes in posture by mapping changes in CoP to changes in LE muscle activations (Figure 1).

The user stood at an erect, nominal stance with baseline (open-loop) stimulation. In this stance, the user stood upright with the feet approximately under the shoulders and each on a separate force plate. The erect, nominal stance is biomechanically defined as the standing posture in which the head, trunk, pelvis, and LEs are aligned as close to vertical as possible in sagittal and coronal planes with minimal to no axial rotation in the coronal plane. The components of the overall CoP position in the AP and ML direction were computed using (equation (1)) and (equation (2)), respectively [17].

(1) $$\begin{array}{c} {\text{CoP}}_{\text{AP}}={\text{CoP}}_{\text{Lx}}*\left(\frac{{\text{F}}_{\text{L}}}{{\text{F}}_{\text{L}}+{\text{F}}_{\text{R}}}\right)+\text{Co}{\text{P}}_{\text{Rx}}*\left(\frac{{\text{F}}_{\text{R}}}{{\text{F}}_{\text{L}}+{\text{F}}_{\text{R}}}\right), \end{array}$$

(2) $$\begin{array}{c} {\text{CoP}}_{\text{ML}}=\text{Co}{\text{P}}_{\text{Ly}}*\left(\frac{{\text{F}}_{\text{L}}}{{\text{F}}_{\text{L}}+{\text{F}}_{\text{R}}}\right)+\text{Co}{\text{P}}_{\text{Ry}}*\left(\frac{{\text{F}}_{\text{R}}}{{\text{F}}_{\text{L}}+{\text{F}}_{\text{R}}}\right), \end{array}$$

where CoP_AP_ and CoP_ML_ are the AP and ML components of the CoP. CoP_Lx_, CoP_Ly_, CoP_Rx_, and CoP_Ry_ are the *x* and *y* components of the CoP, and F_L_ and F_R_ are the vertical reaction forces under the left and right feet, respectively.

As the subject leans away from the erect stance and the CoP moves away from the nominal (erect) position, the resulting changes in AP and ML components are tracked by the PFC via a simple proportional control law. Assuming a linear relationship between changes in the CoP and muscle activation, the changes in activation to be applied to the LE muscles are computed according to (equation 3):

(3) $$\begin{array}{c} \text{Muscle activation}=\text{BPW}+{\text{Gain}}_{i}*\frac{\left(\text{SPW}‐\text{BPW}\right)*{\Delta \text{CoP}}_{i}}{\text{Max }{\text{CoP}}_{\text{iD}}}, \end{array}$$

where BPW is the baseline pulse width value, Gain*_i_* is the proportional gain applied to the AP and ML components of the directions of the overall CoP, SPW is the muscle saturation pulse width value (defined as the maximum above which no additional force is generated), CoP*_i_* is the subject's instantaneous CoP position, and Max CoP_iD_ is the maximum excursion of the path of the object being tracked by the subject's CoP relative to the nominal position. In this equation, *i* is a placeholder for either the AP or ML component of CoP. Thus, ΔCoP*_i_* is the difference between the current CoP position and the nominal CoP position in the AP or ML direction. The gain setting was limited to values between 0 and 1, to prevent the muscle activation from exceeding SPW.

Assuming that posture is adjusted in a slow and quasi-static manner, the PFC targets muscles to provide support, supplying stimulation that is optimal, as determined in [10], for the static position at any time. Previous simulation studies [10, 18] determined that the bilateral GS, GMED, and PA provide support as the postural shifts are elicited away from the erect stance. Based on these findings, the PFC only modulates activation to those muscles. The GS are ankle plantar flexors and were modulated as posture was adjusted forward and backward in the sagittal plane. The TAs are ankle dorsiflexors and were activated at a fixed pulse width to cocontract with the GS, to increase ankle joint stiffness or, as we observed in the current subject, to help mitigate any spasms that might be triggered as posture is adjusted. The GMED and PA are hip ab/adductors, respectively, and were utilized to effect postural shifts in the ML direction. When posture was adjusted leftward, the right GMED and left PA were targeted for activation to support the body against the pull of gravity. Conversely, when posture was adjusted rightward, the left GMED and right PA were targeted for activation. The PFC did not modulate muscle activation to the extensor muscles (right HM, right ES, and bilateral GMX and QD) which had to be maximally stimulated to maintain an erect standing posture.

### 2.3. Visual Feedback

**Figure 2:**
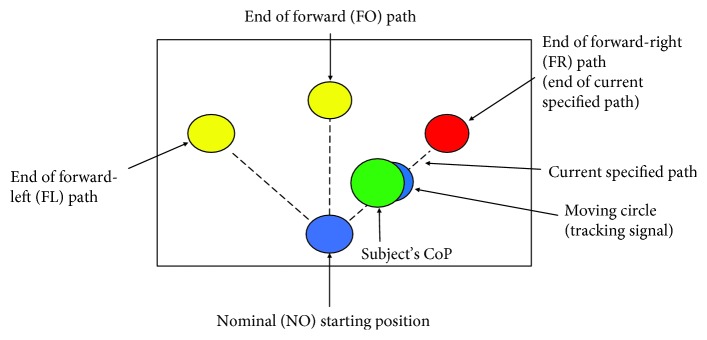
Diagram of the visual feedback display. The subject stood erect at the nominal (NO) starting position and adjusted the overall center of pressure (CoP) position to track the moving circle to the end of the paths defined by the yellow circles. The subject tracked the moving circle along the same path to return to the NO position. Prior to conducting the experiments, the speed of the moving circle and the locations of the endpoints of the paths were tuned to ensure that the subject adjusted posture at a comfortable rate and within reasonable limits of his standing balance. During the experiments, the subject adjusted the overall CoP position (green) to track the moving circle (blue) in the forward (FO), forward-right (FR), and forward-left (FL) directions. As an additional visual cue, the currently specified path is defined by changing the color of its endpoint from yellow to red. In this image, the currently specified path is the one from NO to FR.

To assess the standing performance with respect to maintaining and tracking posture according to a prescribed path, visual feedback of the overall CoP and specified paths were presented on a computer monitor in real time (Figure 2). To ensure that posture was adjusted at a consistent rate, the subject adjusted his CoP to track a circle moving along a straight line on the computer screen. The moving circle traveled at a speed of 20 mm/s to the end of the selected path and returned to the nominal position along the same path. This speed was selected because it was the maximum speed that enabled the subject to adjust CoP in a continuous manner. The length of each of the paths was based on the subject's comfort while leaning forward and diagonally. The endpoints of each path were positioned 40 mm and 48 mm from the nominal starting position in the forward and diagonal directions, respectively.

### 2.4. Posture Follower Controller Tuning

The proportional gain settings in (equation 3) were tuned by hand over several experimental sessions. To determine the optimal settings, the subject stood erect on the force plates with visual feedback and adjusted posture to place his CoP at the location of the endpoints of the paths in the forward and diagonal directions. If the gain settings were too high, activation to the targeted muscles may require the subject to exert higher UE forces to resist the muscle actions. Conversely, if the gain settings were too low, the controller would only nominally modulate activation to the LEs and diminish its potential impact on the posture. The gain values that enabled the subject to comfortably adjust posture to and from the ends of all the paths were implemented in all the subsequent repetitions with the moving circle.

### 2.5. Data Capture

**Figure 3:**
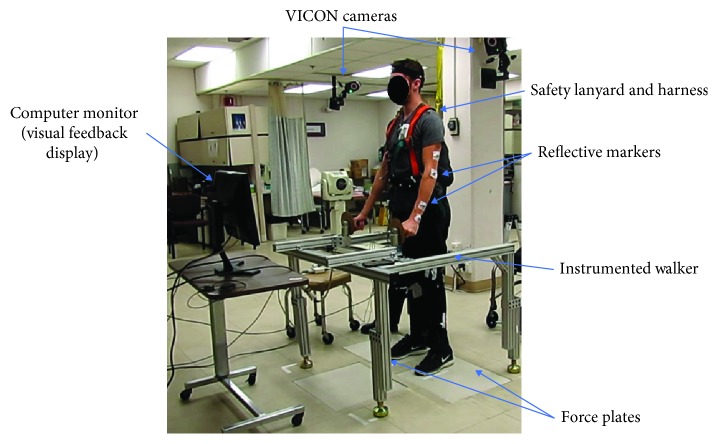
Set-up for experimental evaluation of the controller. The subject stands erect on force plates, while holding onto an instrumented walker and adjusting the overall center of pressure (CoP) position towards the end of paths in the forward and diagonal directions. The subject was provided with visual feedback while adjusting the overall CoP position. Reflective markers were mounted on the subject to track his joint positions as he adjusted posture.

The setup for testing the effects of the PFC is depicted in Figure 3. After activating baseline stimulation to transition from sitting to upright standing, the subject donned a suspension harness (McMaster-Carr Inc., Elmhurst, IL) attached to a lanyard (Guardian Fall Protection, Kent, WA) connected to a hook bolted into the laboratory ceiling decking for safety. The subject stood with his hands on a custom-built adjustable-instrumented walker (80/20, Columbia City, IN), which was adjusted for his height and comfort. The subject also stood with each foot placed on a separate force platform to compute the overall CoP position in the AP and ML directions (equations 1 and 2). Upon settling into a comfortable erect standing position, the locations of the subject's feet were marked with a tape to ensure the same foot placement throughout the experimental session.

A static trial was collected to obtain the UE forces exerted on the walker at the nominal erect standing posture. After instruction and sufficient practice to obviate learning effects, the subject adjusted posture by exerting volitional UE effort on the instrumented walker to ensure his overall CoP tracked the moving circle as it moved in the forward (FO) and diagonal directions (forward-right, FR; forward-left, FL). Five trials were collected, with two repetitions for tracking the circle to the ends of each of the three paths and returning to the nominal erect position completed per trial. The sequence of directions was randomized to avoid systematic error.

### 2.6. Data Analysis

**Figure 4:**
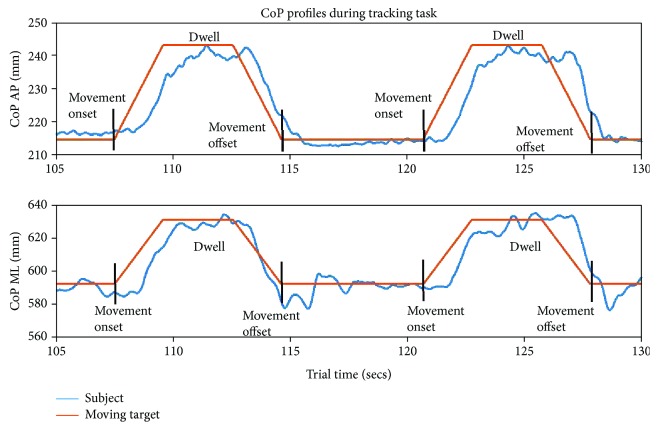
The sample CoP profiles of the subject (blue) and the moving circle (red) of the two consecutive leaning movements during the tracking task. The top panel displays the CoP profiles in the AP direction, while the bottom panel displays the CoP profiles in the ML direction. Each movement is considered a separate repetition, which consists of a movement onset, dwell period, and movement offset. Movement onsets are indicated as the time point in which the moving circle initiates movement from the nominal starting position to the end of the specified path. Upon reaching the end of the specified path, the moving circle dwells there for 3 seconds. When the dwell period ends, the moving circle returns to the nominal position and remains there until it initiates travel along the next path. The first time point at which the moving circle acquires the nominal starting position on the return is the movement offset. Based on the orientation of the laboratory coordinate system, postural adjustments in the forward direction are indicated as CoP_AP_ increasing from the nominal starting position. The postural shifts towards the left are indicated as CoP_ML_ increasing from the nominal. Thus, in both repetitions, the subject was tracking the moving circle in the forward-left direction.

A repetition is distinguished by leaning movement onset and offset (Figure 4). Movement onset was defined as the initial time point where the moving circle departed from the nominal starting position. Upon reaching the end of the path, the moving circle dwelled there for 3 seconds before returning to the nominal starting position. Movement offset was defined as the time point where the moving circle first acquired the nominal starting position on its return.

There were twelve repetitions in which the subject consistently maintained the starting position before movement onset, tracked the moving circle the entire distance to the end, and maintained the same nominal starting position after movement offset. Those repetitions were selected for analysis, and the overall CoP profiles were computed. To obtain the changes in the CoP relative to the value at the nominal position, the starting CoP position was subtracted from the resulting trajectories. UE effort, defined as the maximum resultant UE forces exerted with the PFC, was compared to the values exerted during the erect stance (equation 4).

(4) $$\begin{array}{c} \text{Percent difference}=\frac{{\text{UE}}_{\text{NO}}-\text{U}{\text{E}}_{D}}{{\text{UE}}_{\text{NO}}}*100, \end{array}$$

where UE_NO_ is the maximum UE force exerted at the nominal position, and UE_D_ is the mean maximal UE effort exerted while changing posture along the three directions (FO, FR, and FL). Given the quasi-static nature of the tracking tasks performed in this feasibility study, the 95% prediction ellipse area (PEA) was computed to describe the dispersion of the CoP position in the directions investigated. The 95% prediction ellipse represents a region that contains the center of the points of the postural sway with 95% probability. Schubert and Kirchner [19] recommended the PEA as a standard method of measuring posturographical scatter data, instead of the confidence ellipse. PEA was computed as shown in (equation 5).

(5) $$\begin{array}{c} \text{PEA}=\pi *\chi_{2}^{2}*\sqrt{\det\left(\text{S}\right)}, \end{array}$$

where *χ*_2_^2^ is the inverse of the chi-square cumulative distribution function with 2 degrees of freedom at a fixed probability level (*P* = 95%, *χ*_20.95,2_^2^ ≈ 5.99146), det(S) is the determinant of the Eigenvalues of the sample variance covariance matrix of CoP_AP_ and CoP_ML_. The PEA and the width of each prediction ellipse, a measure of CoP deviation from specified path, quantified the CoP-tracking performance.

## 3. Results

### 3.1. Controller Tuning

When movements were elicited in the forward or diagonal directions at gain settings larger than 0.4 of the changes in CoP_AP_, the modulated activation to the bilateral GS resulted in raising the heels off the ground so the subject stood on his toes. This heel-raising effect was diminished when the gain was set to values below 0.35 and the SPWs of the right and left GS were reduced from 100 *μ*s to 65 *μ*s and from 90 *μ*s to 70 *μ*s, respectively. The tuned SPW for the target muscles are listed in Table 1. When the gain setting on changes in CoP_ML_ was increased to 0.5 (with the tuned gain and SPW settings for CoP shifts in the AP direction), no undesirable changes in posture were observed. Therefore, the PFC gain for ML direction was set at 0.5 for all repetitions.

### 3.2. Controller Actions

**Figure 5:**
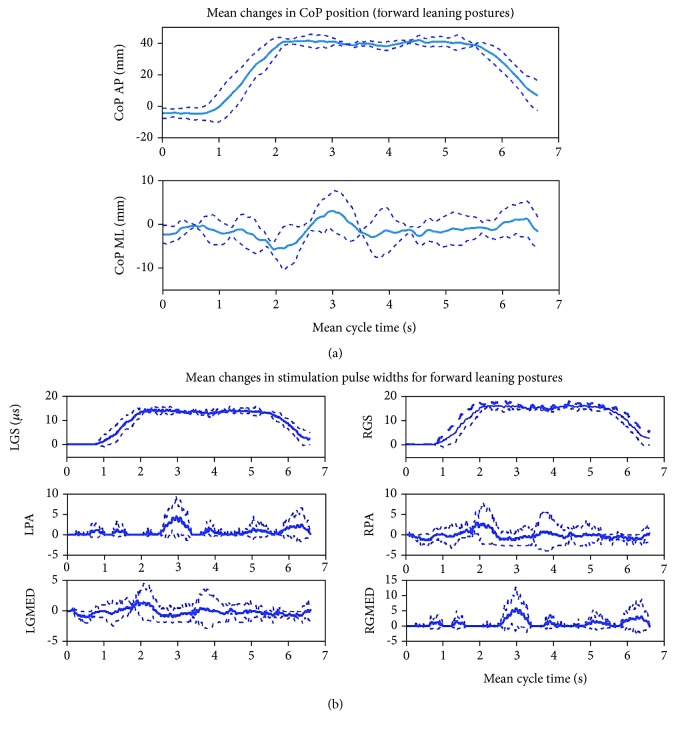
Mean changes across the five trials in (a) the overall CoP position and (b) muscle stimulation pulse widths as posture was shifted in the forward direction. In (a), the mean CoP profiles are presented for the anterior-posterior (AP) direction and the medial-lateral (ML) direction. In (b), the changes in stimulation PWs are presented for the following muscles: LGS (left gastrocnemius), RGS (right gastrocnemius), LTA (left tibialis anterior), RTA (right tibialis anterior), LGMED (left gluteus medius), RGMED (right gluteus medius), LPA (left posterior adductor), and RPA (right posterior adductor). In all plots of the repetitions in the forward direction, the mean profiles are indicated with bold solid lines and (±1) standard deviation is indicated with dashed lines.

The mean changes in CoP trajectories and stimulation pulse widths for leaning postures in the FO direction are represented in Figure 5. After about 1 second, posture began to change from the nominal starting position and arrived at the end of the path after approximately 2 seconds. The primary muscles activated during this movement were the bilateral GS, as the greatest change in the CoP position occurred in the AP direction. The leaning posture was maintained for about 3 seconds, during which, fluctuations in the ML component of CoP were elicited to ensure posture was maintained. These postural adjustments resulted in activation to the left GMED and right PA at the beginning and end of the dwell period. The left PA and right GMED were activated in the middle of the dwell period. This suggests that to maintain the FO leaning posture for this subject, adjustments towards the right were required at the beginning and end of the dwell period. To maintain the FO-leaning posture during the middle of the dwell period, adjustments towards the left were required. During the dwell period, the largest changes in activations were to the bilateral GS, which each reached a maximum of 16 *μ*s. After the 3-second dwell period, posture was adjusted back towards the nominal starting position. Activation to the bilateral GS decreased as the nominal posture was attained. Activations to the bilateral PAs and GMEDs were minimal during this portion of the leaning movement.

### 3.3. Standing Performance

**Figure 6:**
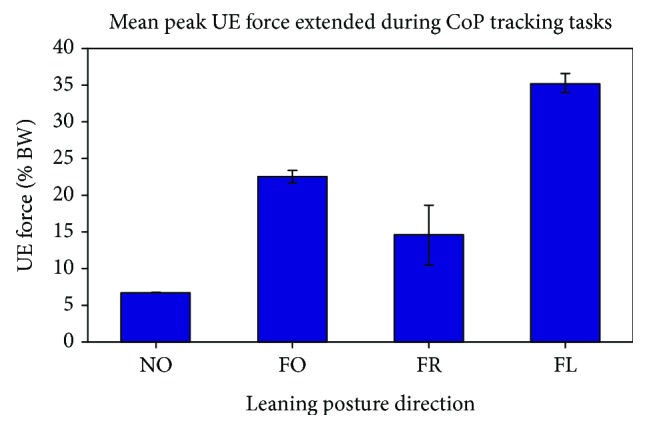
Mean maximum resultant UE force during leaning movements in the forward (FO), forward-right (FR), and forward-left (FL) directions. As a reference, the maximum resultant UE force exerted while the subject stood in the nominal (NO) starting position during a static trial is also displayed. Error bars are included to indicate ±1 standard deviation of measurements across twelve repetitions.

Standing duration was an average of 1 minute and 55 seconds (±6 seconds) per trial. The mean maximum resultant UE forces exerted while changing posture were computed for each leaning direction and normalized as percentage of BW (Figure 6). The mean maximum resultant UE effort exerted while eliciting leaning movements in the FO, FR, and FL directions were 22.5 ± 0.9%BW, 14.6 ± 4.1%BW, and 35.2 ± 1.3%BW, respectively. As a reference, the maximum resultant UE force (6.75% BW) exerted while standing in the nominal (NO) starting position is also displayed. Compared to the maximum resultant UE force exerted at NO, the percent difference in the mean maximum resultant UE effort exerted during leaning movements in the FO, FR, and FL directions was 233.3%, 116.3%, and 421.5%, respectively.

**Figure 7:**
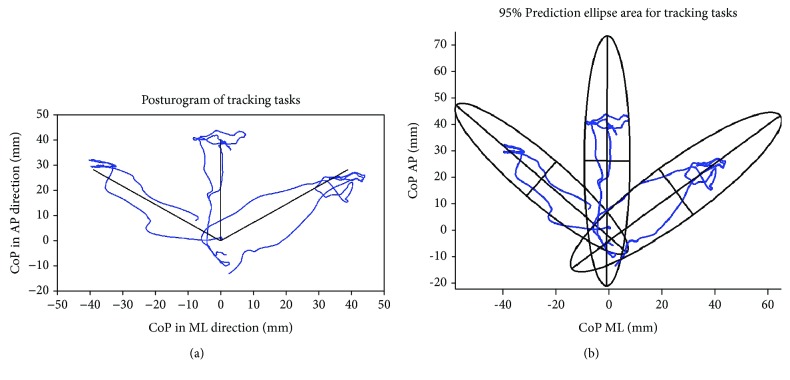
(a) Typical posturogram and (b) 95% prediction ellipses for CoP-tracking tasks in the forward and diagonal directions. The 95% PEA for the leaning movements in the forward direction was 1276.5 mm^2^, 1141.8 mm^2^ in the forward-right direction, and 1645.2 mm^2^ in the forward-left direction. Based on the orientation of the laboratory coordinate system, postural adjustments in the forward direction are indicated as CoP_AP_ increasing from the nominal starting position. Postural shifts towards the left are indicated as CoP_ML_ increasing from the nominal.

CoP excursions in the AP and ML directions during the CoP-tracking task are displayed in Figure 7(a) and are representative of one repetition of leaning movements in each direction. To adjust posture in the FO direction, changes in CoP position in the AP direction were mainly required, with minimal changes to CoP in the ML direction (as also indicated in Figure 5(a)). To lean in the FR direction, posture was adjusted about 10 mm more in the ML direction than the AP direction. While leaning in the FL direction, posture was adjusted about the same distance in the AP and ML directions. However, maintaining posture at the end of each path required adjusting CoP position in both the AP and ML directions. To maintain the leaning postures at the end of the FO and FR paths, adjustments in the CoP position were mainly elicited in the ML direction. To maintain the leaning posture at the end of the FL path, adjustments in the CoP position were elicited in both the AP and ML directions. The mean 95% PEA for leaning movements in the FO, FR, and FL directions were 951.0 ± 341.1 mm^2^, 1095.9 ± 251.2 mm^2^, and 1364.5 ± 688.2 mm^2^, respectively. The 95% PEA was the greatest for leaning movements in the FL direction, suggesting that the overall CoP position deviated from the moving circle when leaning toward the end of the path and returning to NO, as illustrated in Figure 7(a). The prediction ellipses for one repetition of leaning movements in each direction are displayed in Figure 7(b). The 95% PEA for the leaning movement in the FO direction was 1276.5 mm^2^, while the 95% PEA was 1141.8 mm^2^ and 1645.2 mm^2^ for the leaning movement in FR and FL directions, respectively.

**Figure 8:**
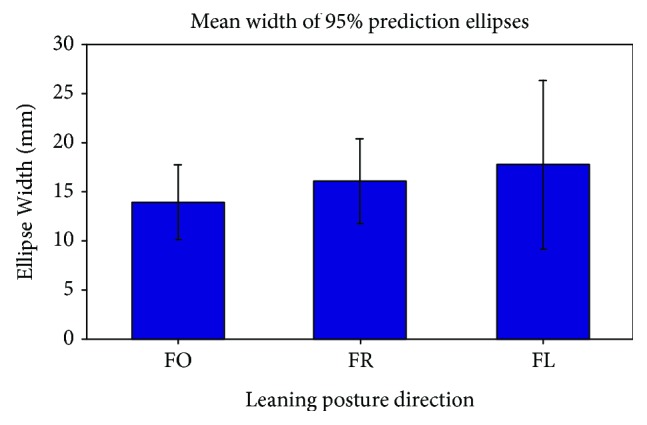
Mean widths of 95% prediction ellipses for CoP-tracking tasks in the forward (FO), forward-right (FR), and forward-left (FL) directions. The error bars are included to indicate ±1 standard deviation of measurements across twelve repetitions.

The mean width of the prediction ellipses (Figure 8) for leaning movements in the FO, FR, and FL directions were 13.9 ± 3.8 mm, 16.1 ± 4.3 mm, and 17.7 ± 8.6 mm, respectively. The FL direction has the largest ellipse width and greatest PEA, further suggesting that greater CoP deviations from the moving circle occurred in that direction.

## 4. Discussion

The aim of this study was to implement the PFC in the laboratory setting and conduct an experimental feasibility test with a standing NP user. This is the first study to our knowledge to investigate the modulation of LE stimulation in a standing NP user as posture is adjusted away from erect stance via a feedback controller. In this study, the feedback signal was the CoP position, which was readily obtained from force plates. As the subject leaned away from an erect stance, the PFC modulated stimulation proportionally according to the desire to effect postural change during the tracking tasks.

Compared to the maximum resultant UE force exerted while the subject stood in the NO position during the static trial (6.75% BW), large percent differences in mean maximum resultant UE effort exerted were observed for all the leaning directions (%difference ≥ 116.3%). In the simulation study [10], the PFC reduced UE effort by an average of 50%, compared with UE effort alone. UE contribution to leaning postures was modeled as simple impedance forces defined as linear functions of the shoulder position. In these experiments, the subject's specific volitional strategy to use the UEs to elicit changes in posture was not controlled. The PFC modulated activation of the paralyzed LE muscles only according to changes in the CoP position. While the movement strategies may vary slightly from trial to trial and condition to condition, the overall strategy implemented was consistent over all the trials. Any variations in UE muscle activation from condition to condition would minimally affect the movement of the CoP and average out over the repeated trials. While the movement strategies may also vary from subject to subject, the user acted at his own control, and it is unlikely that voluntary UE muscle activation patterns would change significantly. No visual differences in the strategy implemented to adjust posture across trials were observed; thus, changes in UE muscle activation were not anticipated. The findings in this experimental study indicate that there are greater demands placed on the UEs while changing posture, suggesting the impedance model may be a highly simplified representation of UE contribution to leaning movements. Thus, future work should explore more accurate representations of the interaction forces between the UEs and the support device during leaning movements. Another potentially confounding assumption in the model was that the feet were fixed to the ground and not allowed to rotate. Thus, the simulation outcomes could freely apply high-activation levels to the ankle plantar flexors without causing the model to fall over. These might have been the reasons for a high reduction in the UE forces in simulation, which was not practicable in real life because of the heel-lifting effect observed for the current study participant.

PEA and ellipse width were computed to determine CoP-tracking deviations. Across all leaning directions, the PEA increased as deviations in CoP tracking occurred (Figure 7). The ellipse width provided an additional measure of CoP tracking, as it described how far the overall CoP deviated from the prescribed straight-line path (Figure 8). The mean peak resultant UE effort, PEA, and ellipse width were all greatest during leaning movements in the FL direction, but were relatively similar for postural changes in the FO and FR directions. These findings may suggest that as the leaning posture deviated from the prescribed path, more UE effort may have been required to readjust posture towards the path. The PFC continually updated stimulation to the LEs as the changes in posture were elicited. The differences in the findings for the FR and FL directions may be attributed to differences in UE strength or the individual differences in the stimulated responses of each muscle, among other issues particular to this subject. Future work will repeat similar experiments with additional subjects.

Lemay et al. [20] conducted the comfortable multidirectional limits of stability test with visual feedback to investigate dynamic postural stability in ambulatory individuals with SCI. They reported CoP_area_, defined by an ellipse fitting the linear distance between the initial and maximal positions of the CoP in each of the eight tested directions (FO, FR, FL, right, left, backward, backward-right, and backward-left as an outcome measure). The average CoP_area_ was 20,181.8 ± 4527.8 mm^2^ compared to 19,332.4 ± 3557.1 mm^2^ in their able-bodied subject group. We hypothesize that the PEAs observed in our study are less than those reported in [20] due to several differences in the experimental design and subject population. First, our experimental design required the subject to stand within an instrumented walker, which limited how far CoP could be adjusted in each direction, while subjects in the Lemay experiments stood with their hands at their sides without the constraint of an enclosure. Second, our experimental design also required that our subject adjusts the CoP by tracking a moving circle at a fixed velocity. Lemay's subjects had 15 seconds to complete the leaning movement at a self-selected speed. Third, our subject was nonambulatory, had no control of his ankle plantar/dorsiflexors without stimulation, and required a support device to stand. The subjects with SCI in the Lemay study were community ambulators who could stand for 5 minutes without a support device (AIS D), and many had near normal walking ability (1.02 m/s) [20]. Thus, they may not be representative of individuals with incomplete SCI. Lemay et al. [20] further state this as a possible reason that there was no statistical difference found in CoP_area_ between the SCI and able-bodied groups. Future work will repeat these experiments with additional subjects to determine PEAs that are more representative of nonambulatory individuals with incomplete SCI.

The CoP position feedback, as measured with force plates, was a practical signal for laboratory-based exploratory experiments with the PFC. However, the long-term goal is to deploy the controller for home use. Force plates limit controller deployment to the laboratory setting, but advances in sensor technology enable the accurate capture of body motion outside of a controlled laboratory environment. Insole-pressure measurement devices are an appealing option for the measurement of CoP, given that the position of the feet on the floor relative to each other are specified. Each time the user stands, it is likely that the location of the feet will differ slightly. This is not a major issue in the laboratory, where the feet can be moved to fixed targets before each experiment. However, for implementation in the uncontrolled environments of the home and community, additional sensors would need to be added to determine the distances between the feet and their orientation before computing the CoP position. The CoM position is a global variable that can be implemented to detect the position of the body each time the user stands as well as to track the dynamic changes in posture as the user prepares for a functional task. Furthermore, the CoM position more accurately reflects the system dynamics and can change without commensurate displacements of the CoP. The CoM position is therefore an ideal parameter for controlling the entire system, particularly for faster movements or to recover from perturbations. Methods to estimate the CoM position from a network of body-mounted inertial measurement units are underdeveloped, and future work will verify such techniques and incorporate them into home-going systems employing the whole-body CoM position as the feedback signal.

A limitation of this study is the length of time the subject could stand during the experiments. Although the subject could stand quietly for 25 minutes at the time of testing, these experiments were more demanding because they entailed multiple repetitions of standing and adjusting posture in the different directions. To minimize fatigue induced by continuous activation of the muscles, the number of repetitions collected was limited, so that the subject's total standing time did not exceed 10 minutes. This is consistent with elapsed standing times with conventional FNS systems [2].

Another limitation to this study is the availability of muscles for control as well as the directions in which the recruited muscles acted. The PFC, as implemented, assumed that the muscles acted independently and exclusively in the sagittal or coronal planes. Future work should explore and exploit the coupling between muscle actions and include cross terms to represent the effects of the GS and TA on ML movement and PA and GMED on AP movement. This involves extending the PFC to act in the generalized coronal plane and modulating all muscles simultaneously irrespective of assumed movement direction (including the postural muscles for hip extension/flexion or trunk extension/lateral bending not adjusted in the current study) to generate the globally optimal patterns of stimulation to realize a movement.

This study sought to determine the experimental feasibility of the PFC, a muscle activation controller that modulated LE activation according to changes in the CoP position, in a recipient of an implanted standing NP. The PFC enabled the subject to assume leaning postures in the FO, FR, and FL directions, by modulating LE muscle activation according to changes in the overall CoP position. More than twice the UE effort as a percentage of quiet standing were required to effect changes in CoP experimentally in this study as predicted from the simulations presented in [10]. CoP-tracking results indicate that all paths presented were successfully tracked, suggesting that the PFC provided the subject with more access to the workspace while standing.

## 5. Conclusions

We have explored the experimental feasibility of the PFC, a CoP-position tracking muscle activation controller with a recipient of an implanted standing NP. This is the first study to our knowledge that investigates feedback control of standing posture to enable user-selected leaning movements away from erect stance in an individual with SCI. As the CoP position was adjusted to track the moving circle along the various paths, the PFC continually updated activation to the user's paralyzed LE musculature. Ellipse areas of the CoP traces indicate that the PFC provided the user with greater access to the standing workspace. Future work will evaluate the controller with the whole-body CoM position as the feedback signal and account for cross-coupling resulting from the anatomical actions of the contracting muscles. This will require the development and evaluation of a model that outputs CoM from data captured from body-mounted sensors and more advanced multidimensional control algorithms.

## Figures and Tables

**Table 1:** Muscle pulse amplitudes and pulse widths for baseline standing. Muscles that were always recruited for baseline standing are indicated with a “P”, while those recruited by the controller are indicated with a “C.” Muscles that were supplemented with surface electrodes are indicated with a “^∗^”.

Muscle	Function	Pulse amplitude (mA)	Baseline standing PW (*μ*s)	Threshold PW (*μ*s)	Saturation PW (*μ*s)
Right gluteus maximus (right GMX)	P	20.0	248	2	250
Right hamstring (right HM)	P	20.0	250	64	250
Left gluteus maximus (left GMX)	P	20.0	145	5	150
Left gluteus medius (left GMED)	C	20.0	61.5	13	110
Right quadratus lumborum (right QL)	—	—	—	—	—
Right erector spinae (right ES)	P	2.1	90	10	125
Left quadratus lumborum (left QL)	—	18.0	0	10	50
Left erector spinae (left ES)	—	18.0	0	35	70
Right quadriceps 1 (right QD 1)	P	0.8	90	30	90
Right quadriceps 2 (right QD 2)	P	0.8	90	24	90
Left quadriceps 1 (left QD 1)	P	0.8	250	64	250
Left quadriceps 2 (left QD 2)	P	0.8	250	48	250
Right quadriceps 3 (right QD 3)	P	0.8	100	32	100
Right posterior adductor (right PA)	C	20.0	86	2	170
Left quadriceps 3 (left QD 3)	P	0.8	250	72	250
Left posterior adductor (left PA)	C	20.0	128.5	7	250
Right gluteus medius (right GMED)	C^∗^	100.0	0	80	250
Right tibialis anterior (right TA)	C^∗^	30.0	0	80	100
Right gastrocnemius (right GS)	C^∗^	100.0	0	20	65
Left tibialis anterior (left TA)	C^∗^	30.0	0	70	90
Left gastrocnemius (left GS)	C^∗^	100.0	0	30	70

## Data Availability

The data used to support the findings of this study are available from the corresponding author upon request.
